# A Report of Two Cases of Sloughing Phagedena

**Published:** 1851-07

**Authors:** Joseph Drew

**Affiliations:** London. House Surgeon to the Manchester Royal Infirmary.


### ARTICLE XXII.

*A Report of Two Cases of Sloughing Phagedena.* By Joseph Drew, M. B., London. House Surgeon to the Manchester Royal Infirmary.

Two cases of sloughing phagedena of the face have recently presented themselves in this infirmary at the same time, and since the best method of treating this affection is still *sub judice*, and knowing that all the old remedies are of little avail, two simultaneous cures through the influence of similar agents cannot but be of interest, and even possess some weight in favor of the means employed.

The first is that of Anthony McDonnell, aged nine, admitted to the wards of this hospital on the 18th of last October. From the statements of the parents, he had been of robust health from infancy until about eighteen month ago, when he became the subject of some febrile affection, but concerning the nature of which they could not particulrrly specify. Six weeks before the date of his admission, he was attacked with acute and continued pain in the left cheek, soon accompanied by a small dark discoloration, which had rapidly increased in size, notwithstanding the treatment of one or two medical men, until it had attained its present dimensions ; the pinched physiognomy and the usual attendant symptoms of this disease were now fully developed, and, on examination, the dark colored slough was found to occupy the whole of the left side of the face, passing irregularly from a point just below the orbital surface of the superior maxillary bone, inwards and downwards along the side of the nose, implicating half of the upper lip, to about half an inch below the angle of the mouth, and backwards and downwards over the substance of the masseter muscle ; the cartilages of the nose were intact ; the immediate edges of the still living parts were deeply reddened, and to all appearances there was no tendency to the formation of a definitive line to its progression. McDonnell was at once put on the best meat diet, ordered daily fifteen grains chlorate of potash in divided doses, and four ounces of sherry wine ; to have the edges of the slough touched all round with strong nitric acid, and a linseed poultice repeated three times a day. Without entering into detail, the remainder of the treatment may be thus summarily stated : for four successive days the acid was freely reapplied, when rapid separation of the slough took place, and subsequently to its complete removal this potent agent was employed at two irregular intervals on a few suspicious points still remaining, so that altogether it was called into use on six different occasions. The fourth day of his admission he was attacked with diarrhea, but it readily yielded to a compound of opium, catechu and chalk mixture. On the cessation of this complication, the chlorate was increased to a scruple, and the wine to six ounces daily ; under these enlarged doses he continued gradually but steadily to improve in appetite and acquire strength, and was made an out-patient on the third of December, or forty-sixth day of treatment. Some time before he left the hospital, it was very evident that a large portion of the superior maxilla would exfoliate, for the ulcerating line of separation was being formed, and bone loosened to an equal extent; and on the seventeenth day after going out, they had progressed so favorably, that evulsion of the parts was performed by the aid of a strong pair of forceps, two out of the four processes of this bone (the alveolar and malar) coming away entire, with two of the milk teeth still firmly implanted in their sockets; since then the surrounding soft parts have been so busy in throwing out plastic organizable matter and contracting, that at the time I now write, the disfigurement is not nearly so great as might have been expected, nor the breach so extensive that it may not be remedied at some future period.

The second case is that of Eliza Lomas, aged six, who was admitted on the 22d October, with an account from her parents that she had been extremely delicate from birth, and that she had suffered from the effects of the usual tribe of infantile diseases in a scrofulous constitution. The history of her present attack very closely resembled that of the boy above related: she had lately undergone some febrile affection; she had been ill a few weeks before presenting herself, and continued to get worse, in spite of medical aid; acute and continuous pain preceded the formation of a slough; and she, too, was now greatly prostrated by the depressive powers of the disease. It did not appear, however, that the morbid process had made any very rapid local progression, for the measurement of the circumference of the dead portion did not exceed that of a wafer of the largest size, extending from a point corresponding to the alveolus of the first molar tooth, completely through the side of the cheek, and implicating, at the same time, more internally, a small portion of the gum; the surrounding edges of the still living parts possessed a deep livid blush, and were found to be tender and somewhat painful.

Precisely similar treatment was put in practice in this case, too: at first fifteen grains of the chlorate, afterwards increased to twenty grains daily, and four ounces of wine, increased to six ounces, the best meat diet, and local application of nitric acid; the latter was used on three successive days before the coming away of the slough, and was esteemed necessary only once afterwards. By the aid of these medicaments she rapidly improved in health, great contraction of the sides of the aperture took place, numerous healthy granulations were thrown out, and by subsequently uniting with others from the implicated portion of gum, this gap, though at first large enough to admit the middle finger, was completely closed, and she went out of the hospital, on the 10th of December, with a slightly sunken and puckered cheek, but in all other respects better than she had ever been before in her life.

On the fourteenth day of her admission I noticed that about three-quarters of an inch in front of, and somewhat above, the original solution of texture, a small, irregularly circular slough, three lines in diameter, had developed itself; and anticipating its tendency, gently tore away the surface, to ascertain the extent; already it had penetrated to a greater depth than the thickness of the cheek, but fortunately, on account of the very oblique direction, without yet implicating any portion of mucus membrane. Its progress was at once arrested by the strong acid, a clean surface was obtained in two days, kindly granulations were thrown out, and it soon healed, without ever opening into the cavity of the mouth.

*Observations.*—Sloughing phagedena of the face, in children, was recognised by the older writers on medicine; but the late Mr. Pearson was the first to bring the subject prominently before the profession, in a short essay, entitled "Canker of the Mouth," and appended to his "First Principles of Surgery," published in 1788. Since his time many men have thought it worthy of their attention; and though considerable additions have been made to the pathology, still it must be confessed that until very lately no advance had taken place in the requisite therapeutics. Contenting myself, however, by referring those readers who may wish to know more particularly concerning the disease to the writings of Mr. Pearson, Dr. H. Hunt, Dr.

Symonds, Dr. Cuming, and Rokitansky, (Sydenham edition,) I will proceed to notice a few of the peculiarities and points of interest in the cases just related.

1st. The respective ages of these little patients or at least the age of one of them, was considerably above the average of those affected with this disease. Mr. Pearson makes the date of liability vary from eighteen months to six or seven years, and Dr. Symonds from two to five years of age; but one of these instances is a girl of six, and the other a boy of nine, being, in the latter, two years beyond the maximum of Mr. Pearson, and four beyond that of Dr. Symonds.

2d. In each case the formation of the dark slough was preceded by violent and long continued suffering, differing, in this repect, with all the authorities I have been able to consult, for by them, it is generally stated, that very little pain accompanies either by development or the progression; but it would appear from these instances, that sometimes, at least, symptoms of acute inflammatory phagedena may be present. Dr. Hall believes that this affection is subject to the same causes of origin as phagedena of any other mucous part at an early age; and truly, there does not appear to be sufficient ground for supposing that it possesses any specific power of action, or that it is different to sloughing phagedena, affecting any part of the body at a more advanced age, for in one, as in the other, disintegrating granules, just before separation, impart their attributes by continuity of texture, to other portions of the surrounding tissue, which then immediately take on similar virulence of action and tendency to death; it will be found, too, that each possesses many like peculiarities, and when subservient to the power of a remedy, will give way to that endowed with similar virtues.

3d. In the course of the disease in the little girl, it was a point of considerable interest, that on the thirteenth day of treatment, a small, secondary slough should have formed, like, to all appearances, the nature of the primary one, yet not originating in the mucous membrane, or implicating it in the smallest degree, but in the skin, most clearly passing from the surface to within. Calling to mind, at the time it was observed that many physicians believed in the contagiousness of a discharge from such sores, I carefully examined all the edges of ulceration, but found that, contrary to their being the appearance of any suspicious points, the whole surface had assumed a most kindly aspect, and taking this in conjunction with the facts, that the freshly diseased spot was situated above and in front of the original slough, and that the parts and face were cleansed at least once a day with warm milk and water, the evidence seemed sufficiently strong to show that the local disease may occasionally begin, *de novo*, in the skin, as advocated by Dr. M. Hall, and that it does not always, as most authors have stated, commence in the mucous membrane or gums. It is true that the precise point of origin of any local departure from health is not a subject for speculation, but a matter of fact, after due observation; still the known correspondence in structure between skin and mucous membrane, and their intimate sympathies in other affections, would lead us to anticipate that a disease might at one time originate in the cuticular, and at another in the mucous tissue, although known by experience to affect after a directly reverse fashion, in the majority of instances.

4th. The termination of each case possesses its own peculiar interest; that of the lad, on account of the intensity of the disease a youthful constitution may be made to support, and the strong efforts made by nature to close a breach of surface so extensive; that of the girl, on account of the very fortunate recovery that may sometimes be expected to ensue, notwithstanding a delicate conformation of body, and a subjection to continued sickness from birth. Both cures will be rendered more complete at some future period, when, by a slight Taliacotian operation, the breach of substance still existing in the first shall have been filled up, and when, in the second, the band of adhesion uniting the cheek to the gum shall have been divided, and the side of the face once more released with a fair chance of regaining its former position.

5th. The profession is indebted to Dr. H. Hunt for the introduction of chlorate of potash as a remedy for cancrum oris; but since he tendered his account of its virtues in 1843, many persons have thought proper to doubt its efficacy, and others have not hesitated to deny it altogether; still, though it may be quite true that the method of its action upon the organism is doubtful, it is only an accumulation of the evidence of experience that can establish or deny its merits as a medicament. When first made use of, it was thought to produce a beneficial influence by the diffusion of some of its contained oxygen; but as soon as it was objected that it might be detected unchanged in the urine of persons taking it, many thought this fact quite sufficient to upset the chemico-physiological theory of its action, without remembering that it had not been shown that the same amount of the salt might be collected from the urine as was administered by the mouth, and consequently not proved that a portion at least might not be acted upon and decomposed. As far, however, as the experience of five cases will enable me to judge, I should not think it advisable to trust to any constitutional treatment alone, any more than in that of sloughing phagedena in a different part of the body. It is essential that the portions of tissue which have taken on diseased action should be thoroughly destroyed, whether by the nitric acid, or other potential agent, or the actual cautery itself, after the fashion recommended by Mr. Obre, appears to be quite immaterial; neither, on the other hand, should I like to trust to local treatment alone, for the gradual death of tissue is merely the consequence of a constitutional disease; and though layer after layer of texture might be destroyed, still the root of the evil would remain untouched. Both methods should be put in practice at the same time; and I am sanguine enough to think that the person who will put his trust in them will find that this disease, by their combination, is as much under the influence of treatment as most other affections to which our bodies are liable.

6th. In applying the nitric acid it is a matter of some importance to instruct the little patient to inspire fully previous to its application, that the lungs may be filled with air, and thus guarded in some degree against the difficulty or even danger that might result from the inhalation of nitrous fumes; and in the course of treatment it will be found of great advantage to separate carefully every slough as soon as formed, using, if necessary, even some little violence for the attainment of this object, on account of thus obviating to a considerable extent the great contamination of the air of inspiration.

